# Development of a novel IGRA assay to test T cell responsiveness to HBV antigens in whole blood of chronic Hepatitis B patients

**DOI:** 10.1186/s12967-015-0513-1

**Published:** 2015-05-13

**Authors:** Werner Dammermann, Frank Bentzien, Eva-Maria Stiel, Claudia Kühne, Sebastian Ullrich, Julian Schulze zur Wiesch, Stefan Lüth

**Affiliations:** Department of Medicine, University Medical Center Hamburg-Eppendorf, Martinistrasse 52, Hamburg, 20246 Germany; Department of Transfusion Medicine, University Medical Center Hamburg-Eppendorf, Martinistrasse 52, Hamburg, 20246 Germany; Department of Anatomy and Experimental Morphology, University Medical Center Hamburg-Eppendorf, Hamburg, Germany; German Center for Infection Research (DZIF), partner site Hamburg, Hamburg, Germany; Heinrich Pette Institute – Leibniz Institute for Experimental Virology, Hamburg, Germany

**Keywords:** Chronic hepatitis B, HBV, Interferon gamma release assay, Cytokine release assay

## Abstract

**Background:**

Interferon gamma release assays (IGRA) have been developed to support easy and fast diagnosis of diseases like tuberculosis, and CMV in transplant patients. IGRAs focus on cellular immunity especially memory T cells and thus also allow rapid screening prior to complex flow cytometric testing. Here, we describe a novel, sensitive whole blood based cytokine release assay capable of assessing T cell responsiveness to HBV antigens in Hepatitis B patients and assessing hepatitis B vaccination status in healthy individuals.

**Methods:**

Seventy two chronic Hepatitis B patients (CHB), 8 acute hepatitis B patients (AHB) and 80 healthy controls (HC) were tested by ELISA for IFNγ- and IL2-secretion in whole blood after challenge with synthetic peptide libraries of hepatitis B core antigen (HBcAg) or hepatitis B surface antigen (HBsAg).

**Results:**

The developed IGRA test reliably differentiated between Hepatitis B patients, vaccinees and unvaccinated healthy controls. Treatment naïve and treated CHB patients showed a weaker IFNγ response to HBcAg (16 ± 5 and 35 ± 28 pg/ml, respectively) compared to the AHB group (82 ± 39 pg/ml), whereas HC remained unresponsive (6 ± 1 pg/ml). IL2 levels after HBcAg challenge were also higher in the AHB group compared to naive and treated CHB as well as HC (47 ± 21 vs. 12 ± 3, 15 ± 10 and 12 ± 9 pg/ml, respectively). HBsAg stimulation led to increased IFNγ and IL2 levels in the AHB group (33 ± 12 and 22 ± 12 pg/ml) and even higher levels in HC due to a high hepatitis B vaccination rate (41 ± 10 and 167 ± 58 pg/ml). Naive and treated CHB patients developed no or only weaker IFNγ or IL2 responses to HBsAg (5 ± 2 and 12 ± 7 pg/ml, for naive CHB, 12 ± 10 and 18 ± 15 pg/ml, for treated CHB). For HC, IL2 release after HBsAg stimulation depicted hepatitis B vaccination status with a diagnostic sensitivity and specificity of 85 % and 90 %.

**Conclusion:**

Our novel whole blood based cytokine release assay constitutes an easy and robust tool for screening HBV specific cellular immunity as alternative to flow cytometry or ELISPOT assays.

**Electronic supplementary material:**

The online version of this article (doi:10.1186/s12967-015-0513-1) contains supplementary material, which is available to authorized users.

## Introduction

More than 240 million individuals are infected with chronic hepatitis B worldwide and are at risk to develop severe liver disease, liver cirrhosis or hepatocellular carcinoma [1]. A prophylactic hepatitis B vaccine has been available for over 3 decades and ensures long-term protection from infection [2]. However, development of a therapeutic vaccine for chronic hepatitis B patients has not met with success so far [3]. It is thought that one reason for lacking efficacy of such a therapeutic vaccine is that HBV-specific T lymphocytes show functional defects and exhaustion and lack proliferation in chronic hepatitis B [4, 5].

Effective prophylactic and therapeutic vaccines depend on strong humoral and cellular immune responses to infectious antigens as part of the vaccine formulation. Whereas in vaccine trials the humoral immune response is analyzed by standard serological assays, *e.g.* ELISA or western blot [6,7], cell-mediated immunity (CMI) is usually assessed by established and technically demanding analytical methods like flow cytometry, intracellular cytokine staining (ICS), ^3^H-thymidine proliferation assays or ELISPOT [5]. Therefore, whole blood based cytokine release assays or interferon gamma release assays (IGRA), respectively, may constitute a robust, easy and cost effective alternative as screening tools in studies of HBV Immunology and HBV vaccination studies.

Here, we describe the establishment of a whole blood based cytokine release assay capable of assessing T cell responsiveness to HBV peptide pools in chronic hepatitis B patients and hepatitis B vaccination status in healthy individuals.

## Methods

### Patient selection

Hepatitis B patients treated at the hepatitis outpatient department of the University Medical Center Hamburg-Eppendorf were enrolled in the study for which all patients gave written consent and which was approved by the Ethics Committee of the Hamburg Chamber of Physicians (PV3941). Patients were stratified according to their clinical course into 3 groups (Table 1) with either NUC treatment naïve chronic Hepatitis B (CHB, NUC treatment naive, n = 40), NUC treated chronic Hepatitis B (CHB, NUC treated, n = 32) or acute hepatitis B (AHB, n = 8). NUC are nucleoside/nucleotide analogues (NUC, tenofovir and/or entecavir) used for HBV specific antiviral treatment. Healthy donors of the blood transfusion service at the University Medical Center Hamburg-Eppendorf were anonymously enrolled in the study as healthy controls (HC, n = 80). Additional clinical data for all patients and HC was provided (Table 1).

**Table 1:** Characteristics of all subjects included in the study

Characteristic					
		HC^b^	AHB^b^	CHB^b^, NUC treatment naive	CHB^b^, NUC treated
n		80	8	40	32
	Male	69 (86.3 %)	4 (50 %)	20 (50 %)	23 (71.9 %)
	Female	11 (13.8 %)	4 (50 %)	20 (50 %)	9 (28.1 %)
Age (yr)^a^		40.6 ± 12.0	45.9 ± 15	43.4 ± 14.9	47.3 ± 11.4
	Male	40.2 ± 12.1	49.7 ± 11.8	41.2 ± 11.6	50.1 ± 11.2
	Female	43.7 ± 11.5	42.2 ± 18.7	45.7 ± 17.6	40.0 ± 8.6
ALT (U/l)^a^		n.d.^c^	1046.0 ± 1035.0	37.7 ± 60.2	42.9 ± 39.3
AST (U/l)^a^		n.d.^c^	610.6 ± 702.5	26.6 ± 27.9	29.5 ± 27.7
HBV-DNA (IU/ml)^a^		n.d.^c^	2.88*10^6^ ± 7.7*10^6^	4.2*10^7^ ± 1.8*10^8^	1.6*10^7^ ± 8.8*10^7^
HBsAg (IU/ml)^a^		n.d.^c^	9153.4 ± 10,378.9	12,588.6 ± 20,958.8	4783.1 ± 6326.3
HBeAg	Negative	n.d.^c^	4 (50 %)	34 (85 %)	24 (77.4 %)
	Positive	n.d.^c^	4 (50 %)	6 (15 %)	7 (22.6 %)
Anti-HBs	Negative	41 (51.2 %)	8 (100 %)	36 (90 %)	32 (100 %)
	Positive	33 (41.3 %)	0 (0 %)	4 (10 %)	0 (0 %)

^a^The data are shown as means ± standard deviations

^b^HC = healthy controls, AHB = acute hepatitis B, CHB = chronic hepatitis B

^c^n.d. = not determined

### Reagents

The recall peptide pool CEFT was purchased from JPT, Germany (#PM-CEFT) and solved in sterile dimethyl sulfoxide (DMSO; #RO/A9941/000100, Th. Geyer, Germany) 25 mg/ml followed by storage at −20 °C. CEFT peptide pool consisted of antigenic peptides from human Cytomegalovirus (HHV-5; CMV), Epstein-Barr virus (HHV-4; EBV), Influenza A and *Clostridium tetani*. This positive control pool contained 27 peptides selected from defined HLA class I and II-restricted T-cell epitopes. Considering the high vaccination frequency against Influenza and *C.tetani* and the high prevalence of CMV and EBV in the general population in Germany recall antigen responses were expected for all patient samples. *Staphylococcus aureus* enterotoxin B superantigen (SEB) was obtained from Sigma Aldrich GmbH, Germany (#S4881) and stored 1 mg/ml in sterile, endotoxin-free H_2_O and −20 °C. Synthetic HBV peptide libraries of HBcAg and HBsAg were purchased from JPT, Germany (#PM-HBV-CP and #PM-HBV-lEP, respectively) and solved in sterile dimethyl sulfoxide (DMSO; #RO/A9941/000100, Th. Geyer, Germany) 50 mg/ml followed by storage at −20 °C. Synthetic HBcAg represented a mix of 44 peptides (15 amino acid length each, 11 aa overlap, peptide scan 15/11) comprising the whole amino acid sequence of HBcAg, the 21.5 kDa capsid protein of HBV (genotype A2 subtype adw2, *UniProt: P0C693*). Synthetic HBsAg represented a mix of 98 peptides (15 amino acid length each, 11 aa overlap, peptide scan 15/11) comprising the whole amino acid sequence of HBsAg, the 43.7 kDa surface protein of HBV (genotype A2 subtype adw2, *UniProt: P17101*).

### Cytokine release assay in whole blood *ex vivo*

Venous blood was collected from hepatitis B patients and HC into sterile 7.5 ml Lithium heparin Monovettes (Sarstedt, Germany). 1 ml of whole blood was then dispensed into sterile, pyrogen free 2 ml tubes with screw caps (Sarstedt, Germany) within 4 h of collection pre-loaded with sterile 0.9 % (w/v) NaCl solution (negative control), SEB (1^st^ positive control), recall CEFT peptide pools (2^nd^ positive control) or HBV peptide pools. In all tubes, glucose (2 mg/mI final concentration; pre-diluted in sterile 0.9 % (w/v) NaCl solution; #HN06.1, Carl Roth, Germany) was used to further enhance cytokine secretion, which had been tested before using various antigens (Additional file 1: Online resource 1).

Thus, whole blood was stimulated with a total volume of 120 μl/ tube of NaCl & glucose (negative control), 120 μl/ tube SEB & glucose (1 μg/ml final concentration after dilution out of stock, 1^st^ positive control), 120 μl/ tube recall antigen CEFT & glucose (10 μg/ml final concentration after dilution out of stock, 2^nd^ positive control) or 120 μl/ tube HBV peptide pools (50 μg/ml final concentration after dilution out of stock). All HBV peptide pools were titrated for maximum cytokine responses in advance. SEB served as a 1^st^ positive control due to its superantigenic properties by cross-linking MHC molecules with T-cell receptors, which proved the general viability of all samples. CEFT was introduced as a 2^nd^ positive control to prove the functionality of antigen presenting cells in all samples.

The tubes were closed and incubated at 37 °C for 24 h. Thereafter, plasma supernatants were aspirated, pooled, stabilized with 0.045 % (w/v) NaN_3_ and stored at −20 °C until assayed for cytokines within the next 7 days. A 5 % (v/v) CO_2_ atmosphere was proven to be unnecessary (data not shown) and yielded results comparable to stimulation in the presence of CO_2_.

### IFNγ and IL2 ELISAs

Detection of total / overall amounts of IFNγ and IL2 in human plasma was conducted using ELISA MAX Deluxe sets from Biolegend, Germany: IFNγ (#430106) and IL2 (#431806). The manufacturer’s Avidin-horseradish peroxidase conjugate was replaced by PolyHRP80 streptavidine conjugate (#SP80C, SDT Reagents, Germany) in order to achieve a tenfold lower limit of detection. Lower limit of detection (Background + 3x S.D.) was generally at 2 – 5 pg/ml for IFNγ and IL2, respectively. Initial dilution of control and test samples was performed described as follows: negative control (NaCl) 1/5, 1^st^ positive control (SEB) 1/2500 for IFNγ and 1/500 for IL2, 2^nd^ positive control (CEFT) 1/50 for IFNγ and 1/25 for IL2, test samples (HBcAg and HBsAg) 1/5. If a test sample’s absorbance value fell outside the maximum standard curve range, these samples were subsequently retested with a tenfold higher dilution, *e.g.* 1/5 → 1/50, 1/500 → 1/5000, 1/2500 → 1/25000. A seven-point standard curve from 1 – 64 pg/ml IFNγ or IL2 was used for quantitation. Standards, controls and test samples were measured in duplicate. The samples were analyzed using Magellan software (version 6.5) equipped on a Tecan M200 plate reader.

### Data analysis

#### Software

The ELISA data was analyzed using SigmaPlot software (Systat Software Inc., version number 12.2) and GraphPad Prism software (Graphpad Software Inc., version number 6.04).

#### Statistical analysis

Descriptive statistics, ANOVA including Tamhane T2 post hoc tests and prior normality tests, unpaired *t*-test and receiver operating characteristic (ROC) analysis were performed using SPSS Statistics software (IBM, version number 22), SigmaPlot software (Systat software Inc., version number 12.2) and GraphPad Prism software (Graphpad Software Inc., version number 6.04). Negative control values were deducted from the peptide induced responses prior to statistical analysis. Intra-assay and inter-assay variability could not be assessed due to limited amounts of whole blood per patient.

## Results

### IFNγ responses to HBcAg in whole blood of CHB patients are reduced compared to AHB patients

**Fig. 1:**
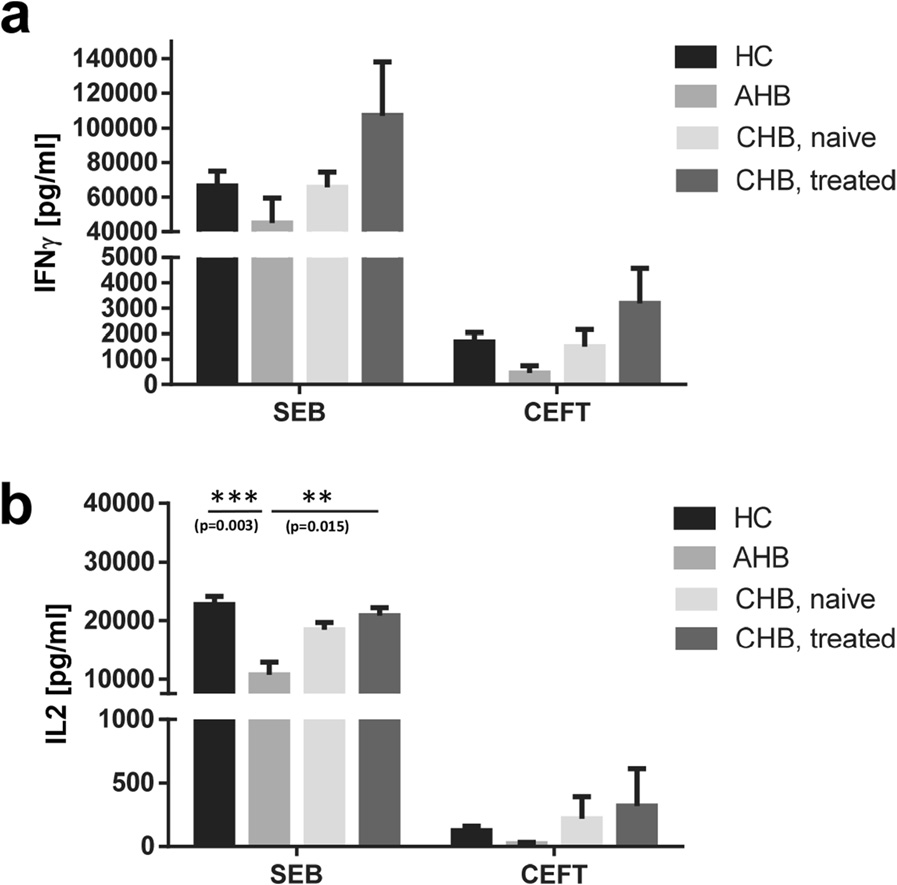
HC and hepatitis B patients show comparable and distinct IFNγ and IL2 responses in whole blood towards control stimulations with SEB and CEFT. n = 80 HC, n = 8 AHB patients, n = 40 NUC treatment naive CHB patients and n = 32 NUC treated patients, for each tested control antigen. Every HC or patient was tested against buffer control, SEB and CEFT. (**a**) IFNγ, (**b**) IL2. Negative control values were deducted from the antigen induced responses. Lower limit of detection (Background + 3x S.D.) was at 2 – 5 pg/ml for IFNγ and IL2. All values are given as mean concentration pg/ml ± S.E.M. ANOVA and Tamhane T2 post hoc tests, following symbol pinpoints significant differences: *. One symbol equals 0.05, two symbols 0.01, three symbols 0.001

Two major HBV proteins, HBcAg and HBsAg, were tested in an HBV specific cytokine release assay with HC and 3 different hepatitis B patient groups: acute, NUC treatment naïve and NUC treated chronic hepatitis B. Stimulation of whole blood with sterile 0.9 % (w/v) NaCl solution (negative control), SEB (1^st^ positive control) and CEFT (2^nd^ positive control) confirmed the viability of all collected samples (Fig. 1. and Table 2).

**Table 2:** Positive and negative controls

Assay	NaCl (negative control)^a^		SEB (1. positive control)^a^		CEFT (2. positive control)^a^		
	IFNγ [pg/ml]	IL2 [pg/ml]	IFNγ [pg/ml]	IL2 [pg/ml]	IFNγ [pg/ml]	IL2 [pg/ml]	N
**Group**							
HC^b^	8 ± 2	2 ± 1	66,537 ± 8485	22,762 ± 1399	1681 ± 361	125 ± 36	80
AHB^b^	15 ± 5	3 ± 1	45,083 ± 14,487	10,737 ± 2148	458 ± 278	22 ± 11	8
CHB^b^, NUC treatment naive	41 ± 22	1 ± 1	65,734 ± 8837	18,439 ± 1244	1490 ± 669	220 ± 173	40
CHB^b^, NUC treated	17 ± 6	2 ± 1	107,200 ± 30,799	20,838 ± 1357	3186 ± 1383	318 ± 293	32

^a^The data are shown as means ± standard error of mean (S.E.M.). SEB and CEFT values are given after deduction of background values (NaCl, negative control)

^b^HC = healthy controls, AHB = acute hepatitis B, CHB = chronic hepatitis B

**Fig. 2:**
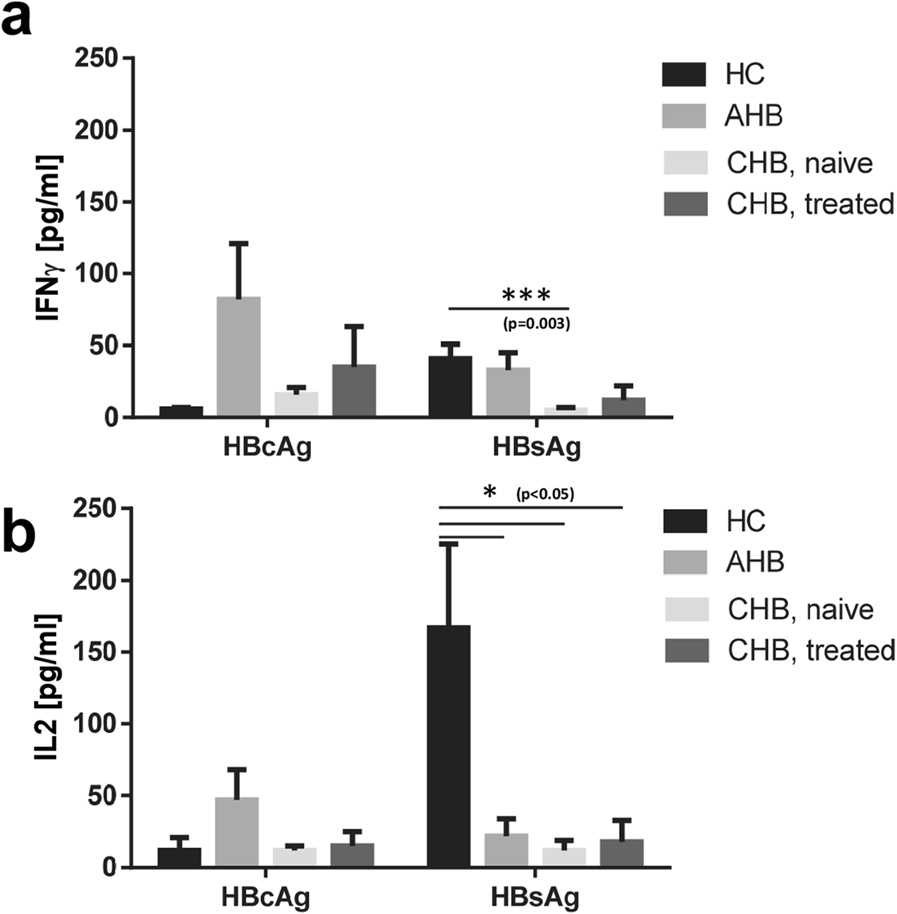
Synthetic HBcAg peptides elicit weak IFNγ and IL2 responses during HBV-specific T cell stimulation in whole blood of CHB patients. n = 80 HC, n = 8 AHB patients, n = 40 NUC treatment naive CHB patients and n = 32 NUC treated patients, for each tested HBV antigen. Every HC or patient was tested against HBV peptide pools specific for HBcAg and HBsAg. (**a**) IFNγ, (**b**) IL2. Negative control values were deducted from the peptide induced responses. Lower limit of detection (Background + 3x S.D.) was at 2 – 5 pg/ml for IFNγ and IL2. All values are given as mean concentration pg/ml ± S.E.M. ANOVA and Tamhane T2 post hoc tests, following symbol pinpoints significant differences: *. One symbol equals 0.05, two symbols 0.01, three symbols 0.001

Synthetic HBcAg-specific peptides elicited higher IFNγ responses in AHB than treatment naïve and treated CHB patients (Fig. 2a, Table 3; 82 ± 39 vs. 16 ± 5 and 35 ± 28 pg/ml; p = 0.78 and p = 0.98). HC did not show any reaction towards HBcAg in whole blood compared to AHB patients (6 ± 1 vs. 82 ± 39 pg/ml, p = 0.62). Synthetic HBsAg peptides induced comparable IFNγ responses in HC and AHB patients (Fig. 2a; 41 ± 10 vs. 33 ± 12 pg/ml; p = 0.99), but weaker responses in naïve as well as treated CHB patients (5 ± 2 and 12 ± 10 pg/ml; naïve CHB vs. HC p = 0.003 and naïve CHB vs. AHB p = 0.46; treated CHB vs. HC p = 0.28 and treated CHB vs. AHB p = 0.9).

**Table 3:** HBcAg and HBsAg stimulations

Assay	HBcAg^a^		HBsAg^a^		
	IFNγ [pg/ml]	IL2 [pg/ml]	IFNγ [pg/ml]	IL2 [pg/ml]	N
**Group**					
HC^b^	6 ± 1	12 ± 9	41 ± 10	167 ± 58	80
AHB^b^	82 ± 39	47 ± 21	33 ± 12	22 ± 12	8
CHB^b^, NUC treatment naive	16 ± 5	12 ± 3	5 ± 2	12 ± 7	40
CHB^b^, NUC treated	35 ± 28	15 ± 10	12 ± 10	18 ± 15	32

^a^The data are shown as means ± standard error of mean (S.E.M.). HBcAg and HBsAg values are given after deduction of background values (NaCl, negative control)

^b^HC = healthy controls, AHB = acute hepatitis B, CHB = chronic hepatitis B

IL2 responses were generally comparable to IFNγ responses regarding total cytokine amounts (Fig. 2b). HBcAg induced IL2 synthesis in AHB (47 ± 21 pg/ml), but not naïve and treated CHB patients as well as HC (12 ± 3, 15 ± 10 and 12 ± 9 pg/ml; p = 0.78, p = 0.89 and p = 0.82). HBsAg elicited significantly higher IL2 responses in HC (167 ± 58 pg/ml) compared to AHB, naïve and treated CHB patients (22 ± 12, 12 ± 7 and 18 ± 15 pg/m; p = 0.05, p = 0.03 and p = 0.04).

Taken together, cytokine release assays using HBcAg stimulation in whole blood are able to depict the (hypo-) responsiveness of HBV-specific T cells in CHB patients.

### HBeAg + CHB patients show weaker cytokine responses to HBcAg and HBsAg than HBeAg- CHB patients

**Fig. 3:**
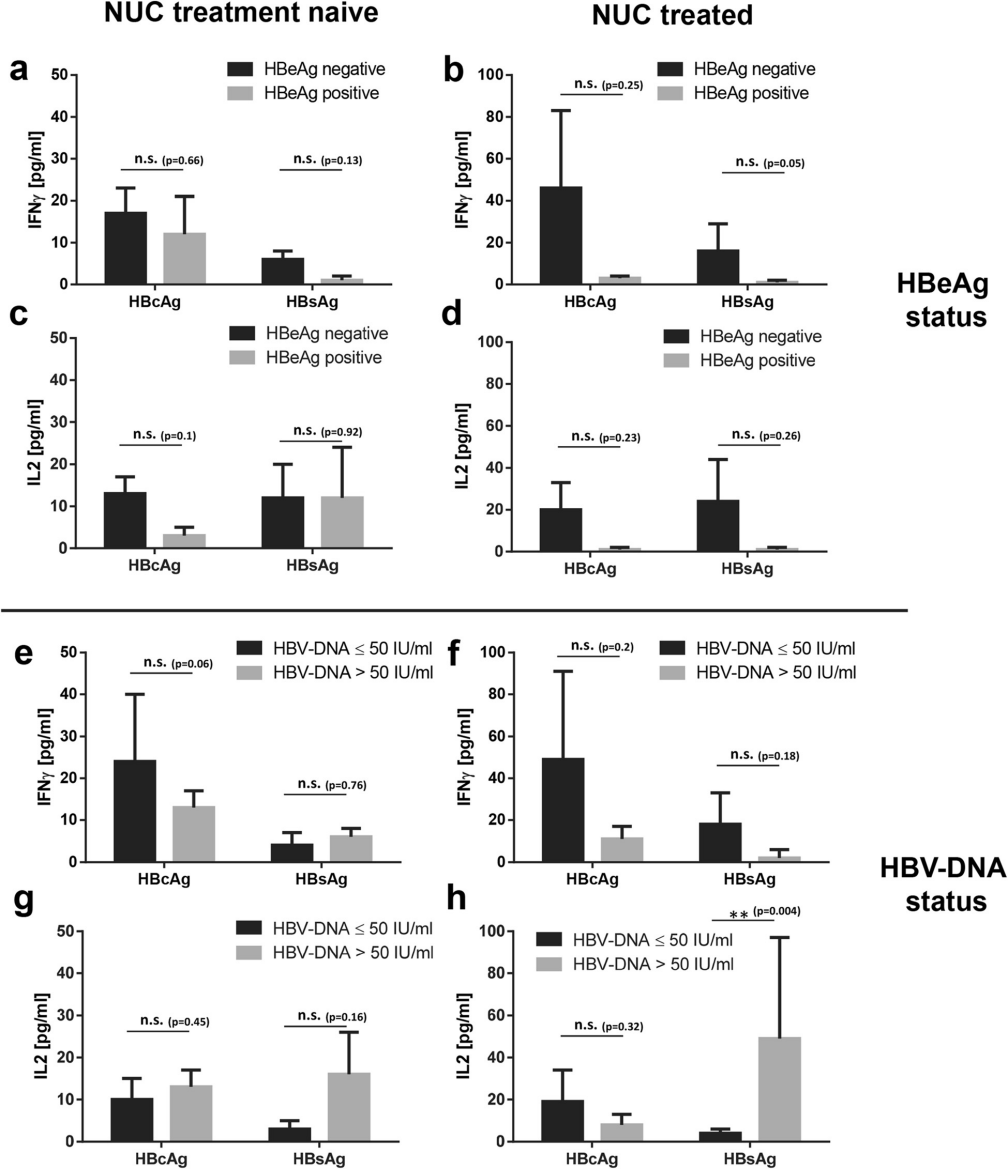
HBeAg + CHB patients show weaker cytokine responses against HBcAg and HBsAg than HBeAg- CHB patients. (**a**) – (**d**) n = 40 NUC treatment naïve CHB patients with n = 6 HBeAg + and n = 34 HBeAg-. n = 32 NUC treated CHB patients, with n = 6 HBeAg + and n = 26 HBeAg-. (**e**) – (**h**) n = 40 NUC treatment naïve CHB patients with n = 10 HBV-DNA ≤50 IU/ml and n = 30 HBV-DNA >50 IU/ml. n = 32 NUC treated CHB patients, with n = 22 HBV-DNA ≤50 IU/ml and n = 10 HBV-DNA >50 IU/ml. Every patient was tested against HBV antigens HBcAg and HBsAg. (**a**), (**b**), (**e**) and (**f**) IFNγ, (**c**), (**d**), (**g**) and (**h**) IL2, respectively. Negative control values were deducted from the antigen induced responses. Lower limit of detection (background + 3x S.D.) was at 2 – 5 pg/ml for IFNγ and IL2. All values are given as mean concentration pg/ml ± S.E.M. Unpaired *t*-test, following symbol pinpoints significant differences: *. One symbol equals 0.05, two symbols 0.01, three symbols 0.001, n.s. not significant

All NUC treatment naïve and NUC treated 72 CHB patients were stratified according to HBeAg status as well as HBV-DNA status and subsequently tested for IFNγ- and IL2-secretion in whole blood after challenge with synthetic HBcAg- and HBsAg-specific peptides by ELISA. Stimulation with HBcAg-specific peptides led to higher IFNγ concentrations in plasma of treated, but not naive HBeAg- CHB patients compared to HBeAg + patients (Fig. 3a - b; 17 ± 6 vs. 12 ± 9 pg/ml for naïve CHB; 46 ± 37 vs. 3 ± 1 pg/ml for treated CHB, respectively). Stimulation with HBsAg-specific peptides was also more prominent in treated, but not naive HBeAg- CHB patients compared to HBeAg + patients (6 ± 2 vs. 1 ± 1 pg/ml for naïve CHB and 16 ± 13 vs. 1 ± 1 pg/ml for treated CHB, respectively). IL2 responses yielded a comparable pattern with stronger cytokine production in plasma of HBeAg- CHB patients compared to HBeAg + patients: treated HBeAg- CHB patients gave higher IL2 levels than HBeAg + CHB patients (Fig. 3d; 20 ± 13 vs. 1 ± 1 pg/ml for HBcAg and 24 ± 20 vs. 1 ± 1 pg/ml for HBsAg, respectively), whereas effects of HBcAg and HBsAg stimulation on IL2 levels in plasma of naïve CHB patients were negligible (Fig. 3c).

Regarding HBV-DNA status we looked for effects of viral load on the cytokine responses directed against HBcAg and HBsAg, but unlike HBeAg status viral load did not seem to influence cytokine production in a similar fashion: low HBV-DNA titers ≤ 50 IU/ml did not always coincide with higher cytokine levels after HBcAg or HBsAg stimulation in treatment naïve and NUC treated CHB patients (Fig. 3e – h).

In conclusion, HBeAg + treatment naïve and NUC treated CHB patients showed the weakest cytokine responses to HBV antigen-specific peptide challenge of all hepatitis B patient groups.

### Treatment naïve CHB patients show comparable cytokine responses to HBcAg and HBsAg compared to NUC treated CHB patients in whole blood

72 CHB patients were stratified according to treatment status with nucleoside/nucleotide analogues (NUC, tenofovir and/or entecavir) and subsequently tested for IFNγ- and IL2-secretion in whole blood after challenge with synthetic HBcAg and HBsAg peptides by ELISA.

**Fig. 4:**
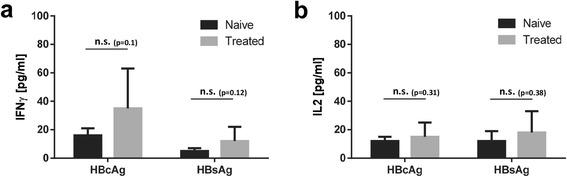
NUC treated CHB patients show comparable cytokine responses to HBcAg and HBsAg compared to treatment naïve CHB patients in whole blood. Lower limit of detection (Background + 3x S.D.) was at 2 – 5 pg/ml for IFNγ and IL2. n = 72 CHB patients, whereas n = 40 NUC treatment naive and n = 32 NUC treated. Every patient was tested against HBV antigens HBcAg and HBsAg. (**a**) IFNγ, (**b**) IL2. Negative control values were deducted from the antigen induced responses. All values are given as mean concentration pg/ml ± S.E.M. Unpaired *t*-test, following symbol pinpoints significant differences: *. One symbol equals 0.05, two symbols 0.01, three symbols 0.001, n.s. not significant

HBcAg and HBsAg stimulation did not result in significant differences regarding IFNγ concentrations in plasma of treatment naïve patients compared to NUC treated patients (Fig. 4a; 16 ± 5 vs. 35 ± 28 pg/ml for HBcAg and 5 ± 2 vs. 12 ± 10 pg/ml for HBsAg, respectively). Likewise, IL2 responses to HBcAg and HBsAg in whole blood yielded no significant differences in view to treatment status (Fig. 4b; 12 ± 3 vs. 15 ± 1 pg/ml for HBcAg and 12 ± 10 vs. 18 ± 15 pg/ml for HBsAg, respectively).

NUC treatment had no significant positive effect on cytokine release in whole blood after stimulation with HBcAg and HBsAg-specifc peptides *ex vivo*.

### IL2 responses to HBsAg in HC correlate with hepatitis B vaccination status

**Fig. 5:**
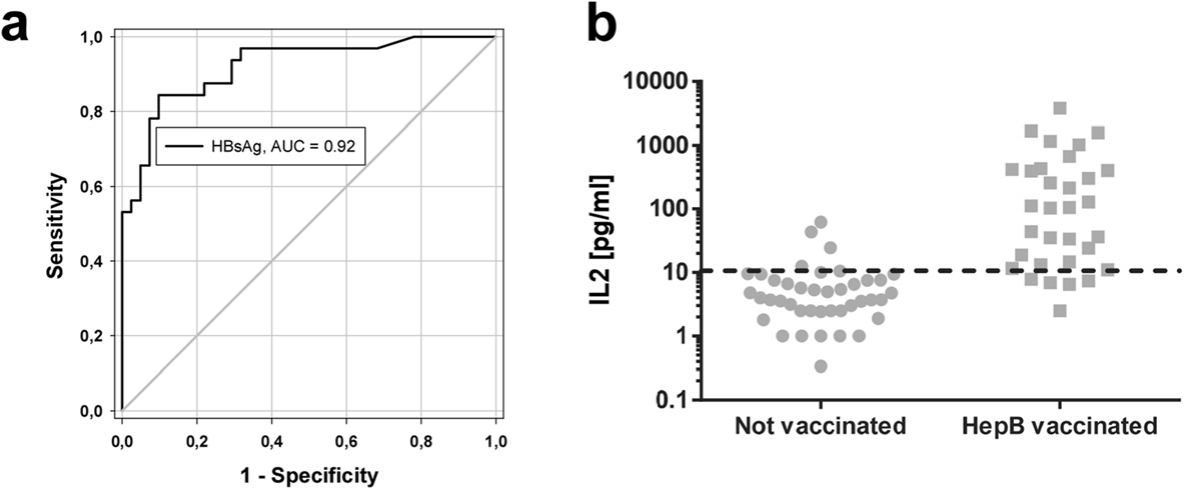
IL2 responses to HBsAg in HC correlate with hepatitis B vaccination status. (**a**) Receiver-operating-characteristic (ROC) curve. AUC, area under the curve. (**b**) Scatter plot of IL2 release of HepB vaccinated and not vaccinated HC after HBsAg stimulation of whole blood. Negative control values were deducted from the antigen induced responses. Dotted line indicates IL2 cut-off at 11 pg/ml. n = 74 HC, whereas n = 33 were anti-HBs positive and n = 41 anti-HBs negative

**Fig. 6:**
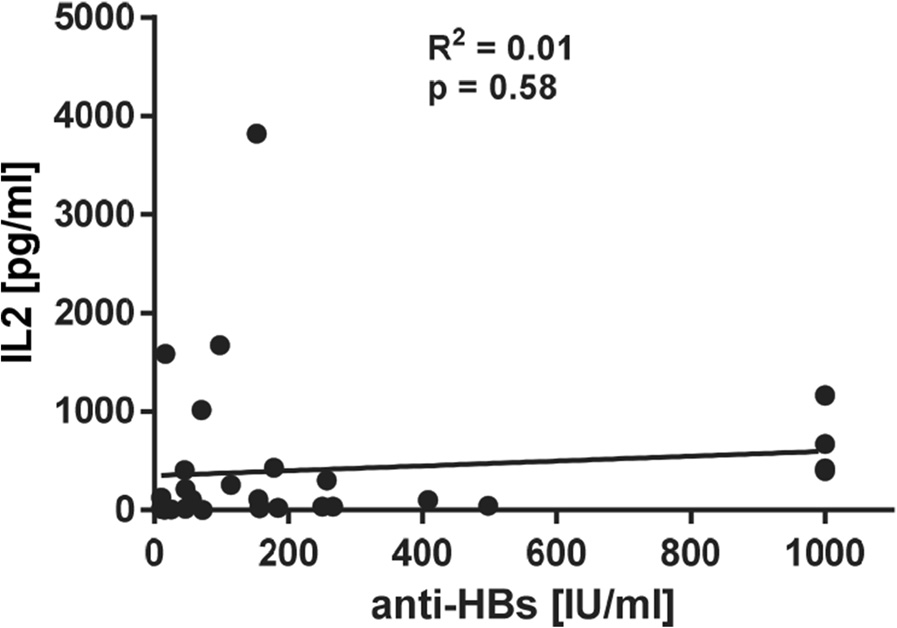
Anti-HBs titers and strength of IL2 response do not correlate in hepatitis B vaccinated HC. Scatter plot of IL2 release of HepB vaccinated HC after HBsAg stimulation vs. anti-HBs titers. Negative control values were deducted from the antigen induced responses. Continuous line indicates linear regression and Pearson correlation. R^2^: coefficient of determination

HBsAg constitutes the single main antigen in hepatitis B vaccine formulations. Thus, 74 HC with known hepatitis B vaccination status were also analyzed regarding their HBsAg-dependent IFNγ and IL2 release. 33/74 HC possessed a positive anti-HBs antibody titer and 41/74 were anti-HBs negative. Whereas IFNγ responses were negligible, IL2 values reached marked heights if the tested individual had been successfully vaccinated (Fig. 5b), but these IL2 levels did not correlate with the corresponding anti-HBs antibody titer (Fig. 6). To determine the cut-off value of our newly developed assay, we performed a ROC analysis of the IL2 readings (Fig. 5a). This HBV specific cytokine release assay with HBsAg-specific peptides using IL2 release as the primary readout (cut-off = 11 pg/ml IL2) reached 85 % diagnostic sensitivity and 90 % diagnostic specificity. The corresponding AUC value was at 0.92. Using the cut-off defined by the ROC analysis, as shown in Fig. 4, 28 (of 33) vaccinated HC showed positive scores, while 4 (of 41) not vaccinated HC slightly exceeded the cut-off value (Fig. 5b).

## Discussion

### Cytokine release assays using whole blood depict (hypo-) responsiveness of HBV-specific T cells in CHB patients

The group of Boni *et al.* delivered key data regarding the HBV specific cellular immune status under treatment with first (lamivudine) or second generation drugs (tenofovir and entecavir) in consecutive and longitudinal clinical studies on HBV specific cellular immunity in CHB patients [4, 5]. In most cases a restoration of anti-viral T cell responses could be observed after *in vitro* expansion, but not directly *ex vivo* [4, 5].

Our current results mirror these findings, since no significant recovery of the cytokine responses to HBcAg and HBsAg could be observed in NUC treated CHB patients in whole blood *ex vivo*. Interestingly, we detected the strongest IFNγ and IL2 responses to HBcAg stimulation in a small pilot group of resolved hepatitis B patients (109 ± 27 and 309 ± 143 pg/ml, n = 3) that we measured for exploratory reasons compared to all other groups, whereas the IL2 response to HBsAg was negligible in contrast to HC (35 ± 4 vs 165 ± 58 pg/ml). It will be intriguing to further analyze this group of patients in follow-up studies *i.e.* to understand in how far the size of the immune response in our assays can predict patients that are at risk of HBV reactivation under immune suppression.

This suppressed T cell state is ongoing even after initiating successful treatment probably due to initially high viral or antigen load and might partially explain why clinical trials with therapeutic hepatitis B vaccines and anti-viral drugs have not been effective so far, whereas treatment safety was generally achieved [8–11]. In these studies, HBV-specific cell mediated immunity and especially the T cell (hypo)-responsiveness of CHB patients was analyzed by established techniques, *e.g.* flow cytometry, intracellular cytokine staining (ICS), ^3^H-thymidine proliferation assays or ELISPOT.

We have demonstrated the suitability of whole blood based cytokine release assays to analyze T cell (hypo-) responsiveness in CHB patients and were even able to differentiate the suppressed T cell state further into HBeAg + CHB patients with stronger suppression than HBeAg- CHB patients. Thus, we propose our protocol as an additional easy-to-use, cost efficient and robust tool for future therapeutic hepatitis B vaccination studies.

### IL2 responses after HBsAg-specific peptide stimulation allow assessment of Hepatitis B vaccination status in HC

Another important aspect of hepatitis B vaccination studies is the emergence of non- and low-responders to vaccination and of previously successfully vaccinated subjects who lost their protective anti-HBs titers over time [12, 13]. In these cases the question arises whether hepatitis B immunity is completely absent with no humoral and cellular immunologic memory or only partial, namely with hepatitis B specific cellular immunity present. The group of Bauer and Jilg proved the presence of significant numbers of HBsAg-specific memory T and B cells in a group of 15 healthy individuals who were successfully vaccinated but had lost anti-HBs titers [13]. A different study used a prototypic third generation HBV vaccine, Sci-B-Vac™, containing small S, PreS1 and PreS2 antigens to trigger cellular and humoral immunity in healthy individuals who failed immunization with conventional vaccines [12]. 15 non-responders and 6 low-responders were included. After three vaccinations, 20/21 subjects developed protective anti-HBs titers ≥10 IU/l, whereas 8/15 non-responders and 5/6 low-responders showed HBsAg-specific T cell immunity using proliferation assays and IFNγ release assays.

In these studies ^3^H-thymidine proliferation assays or IFNγ ELISPOT assays were used to analyze HBV-specific cell-mediated immunity. Both assay types represent well-established techniques using lymphocytes isolated from peripheral blood (PBMCs). However, the direct use of whole blood *ex vivo* and the cytokine IL2 as a novel T cell readout marker might be an attractive alternative in case of future hepatitis B vaccination studies. Our results clearly depicted the hepatitis B vaccination status in healthy individuals and thus should be considered equal to the aforementioned techniques. Interestingly, a strong IL2 response was not mirrored by an equally high antibody titer thus adding valuable information on the immune status. Integrating both humoral and cellular data potentially could give us critical insights regarding overall humoral and cellular immunity towards HBV.

We propose our assay as fast and cost-effective tool for initial screenings of specimen to identify specific candidates which could then subsequently be tested by more technically demanding analytical methods like flow cytometry or ELISPOT.

## Conclusion

We have established a protocol which is capable of analyzing the responsiveness of HBV-specific T cells in patients with chronic hepatitis B infection using whole blood directly for testing without further sample preparation. In addition, we are able to assess the hepatitis B vaccination status of healthy blood donors on the cellular immunity level.

This novel IGRA constitutes an additional easy-to-use, cost-efficient and robust tool for screening HBV specific cellular immunity alone or in addition to other more technically demanding follow-up analytical methods like flow cytometry or ELISPOT.

## Additional file

Additional file 1:
**Online Resource 1 Glucose supplementation to whole blood from HC enhances cytokine secretion during antigen responses.** Stimulations were performed with (a) & (d) VZV antigen peptide pools gE and IE63 (b) & (e) mumps virus lysate or (c) & (f) glucose only. Lower limit of detection (background + 3x S.D.) was at 2 – 5 pg/ml for IFNγ and IL2. Only varicella zoster virus (VZV) and mumps virus seropositive HC are shown. All values are given as single concentration pg/ml. Blue lines indicate median concentrations. Reproducibility was ensured by applying every antigen in two independent experiments with n = 3+ HC per experiment.
