# Supplementary figures and images for: Development of a novel IGRA assay to test T cell responsiveness to HBV antigens in whole blood of chronic Hepatitis B patients
Source: J Transl Med. 2015 May 13;13:157. doi: 10.1186/s12967-015-0513-1 (PMC4465460; doi:10.1186/s12967-015-0513-1)

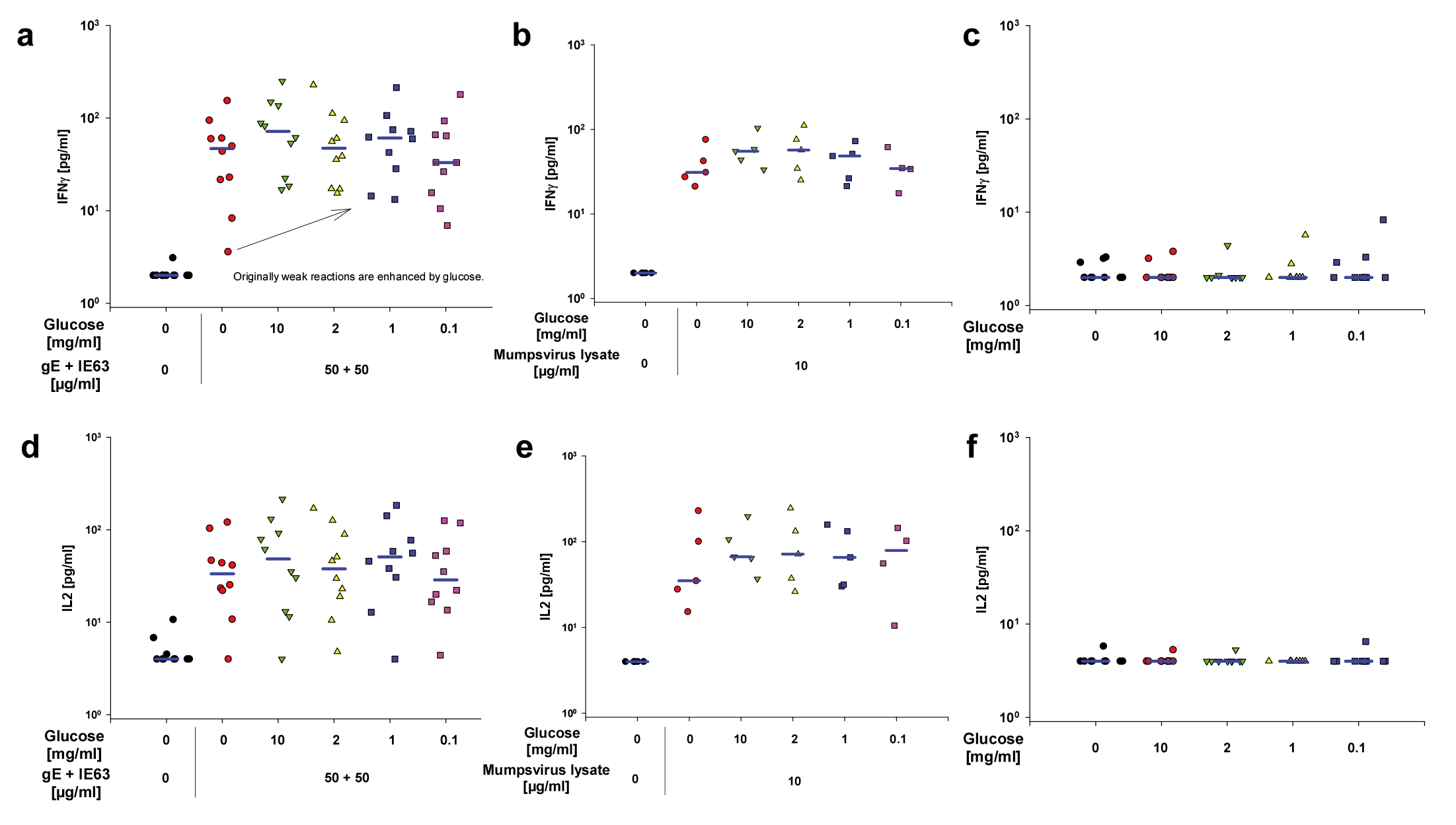

Supplement: Additional file 1: — Online Resource 1 Glucose supplementation to whole blood from HC enhances cytokine secretion during antigen responses. Stimulations were performed with (a) & (d) VZV antigen peptide pools gE and IE63 (b) & (e) mumps virus lysate or (c) & (f) glucose only. Lower limit of detection (background + 3x S.D.) was at 2 – 5 pg/ml for IFNγ and IL2. Only varicella zoster virus (VZV) and mumps virus seropositive HC are shown. All values are given as single concentration pg/ml. Blue lines indicate median concentrations. Reproducibility was ensured by applying every antigen in two independent experiments with n = 3+ HC per experiment. [file 12967_2015_513_MOESM1_ESM.tiff]
